# The Effect of Targeted Temperature Therapy on Antioxidant Levels in Patients With Spontaneous Circulation After Cardiac Arrest

**DOI:** 10.7759/cureus.61578

**Published:** 2024-06-03

**Authors:** Veysi Yazar, Orhan Binici, Mahmut A Karahan, Mehmet B Bilsel, Veli F Pehlivan

**Affiliations:** 1 Anesthesiology and Reanimation, Mehmet Akif Inan Training and Research Hospital, Şanlıurfa, TUR; 2 Anesthesiology and Critical Care, Harran University, Şanlıurfa, TUR; 3 Anesthesiology, Harran University, Şanlıurfa, TUR

**Keywords:** nrf-2, oxidative stress index, total oxidant level, total antioxidant capacity, targeted temperature therapy, cardiopulmonary resuscitation, cardiac arrest

## Abstract

Introduction

In this study, we aimed to measure the change in total antioxidant status (TAS), total oxidant stress (TOS), oxidative stress index (OSI), and nuclear factor erythroid 2 (Nrf-2) levels during the treatment period in patients who restored spontaneous circulation return after cardiac arrest with targeted temperature management (TTM) therapy in our hospital.

Methods

The study included 36 patients who were hospitalized in the anesthesia intensive care unit and coronary intensive care unit of our hospital and were treated with TTM therapy after cardiac arrest. TAS, TOS, OSI, and Nrf-2 levels were measured at 0 (beginning), 12, 24, and 48 (end) hours of TTM therapy.

Results

The mean age of the patients participating in the study was 54.25±17.10. TAS and TOS levels decreased gradually during TTM therapy, but statistically significant decrease was observed at the end of the hour. When Nrf-2 and OSI levels were evaluated, it was found that no statistically significant difference was observed during the TTM therapy.

Conclusion

In our study, the oxidant-antioxidant balance was preserved in patients who received TTM therapy after cardiac arrest. We predict TTM therapy is effective on oxidative stress after cardiac arrest and should be applied for at least 48 hours.

## Introduction

Cardiac arrest (CA) is the sudden, unexpected cessation of circulation and respiratory function due to any cause. It is characterized by the absence of consciousness, absence of pulse, and cessation of breathing [1,2]. Despite all the studies conducted to date, CA remains a public health problem leading to high mortality and morbidity and is one of the leading causes of death in many countries worldwide [3].

Cardiopulmonary resuscitation (CPR) encompasses decisions and actions aimed at restoring spontaneous circulation. CPR is not just an application but also an emergency situation that requires decision-making. Starting CPR promptly and maintaining high-quality CPR is important to achieve a favorable outcome, as it can lead to irreversible brain damage [4].

Ischemia is the inability to reach enough oxygen to the tissues. After ischemia, cell death occurs in the tissue. The reperfusion event required to eliminate the harmful effects of ischemia and clear accumulated toxic metabolites causes much greater damage than the ischemia itself [5]. Hypoxia and ischemia-reperfusion injuries occurring during CA cause damage to multiple organs. Therefore, post-cardiac arrest care involves assessing and mitigating the damage caused by ischemia-reperfusion in multiple organ systems, determining the cause of CA, and treating it [6].

One of the most significant problems encountered in patients with return of spontaneous circulation (ROSC) after CA is neuronal damage during the post-ischemic period, which is associated with the formation of oxidative stress. Oxidative stress induced by ischemia-reperfusion is one of the main mechanisms of tissue damage following cardiac arrest. A decrease in antioxidant defense may contribute to ischemia-reperfusion injury. Hypothermia treatment may improve tissue damage in post-cardiac arrest patients [7].

The reason why the application of targeted temperature management (TTM) in CA is emphasized today is to ensure that patients recover neurologically without sequelae after resuscitation and to try to increase life expectancy and quality of life. This is because hypothermia is recommended as an effective method to prevent certain chemical reactions that occur in conjunction with reperfusion injury after cardiac arrest and to suppress cerebral metabolic activity [8].

In this study, we aimed to measure oxidative stress by assessing changes in total antioxidant status (TAS), total oxidative stress (TOS), oxidative stress index (OSI), and nuclear factor E2-related factor 2 (Nrf-2) levels during the treatment period in patients who achieved ROSC after CA and underwent TTM in our hospital. We aimed to determine the effects of TTM on this stress.

## Materials and methods

Patient selection

Between April 30, 2020, and March 25, 2021, a total of 40 patients who were admitted to the Harran University Faculty of Medicine's anesthesia intensive care unit and cardiology intensive care unit and who underwent TTM after cardiac arrest were included in the study. The local ethics committee approved the study design (date: 05.10.2020, no: 20117), and informed consent was obtained from the patient's relatives. We calculated the sample size based on the results of the first 15 patients in the study. Taking into account these differences and assuming a two-tailed alpha value of 0.05 (sensitivity 95%) and a beta value of 0.20 (study power 80%, effect size 0.53), we determined that a minimum of 40 patients were required for this study (G Power 3 power analysis program).

Inclusion criteria

Patients between the ages of 18 and 75 who achieved ROSC after in-hospital or out-of-hospital cardiac arrest within the first six hours after cardiac arrest and were admitted to Harran University Hospital's anesthesia intensive care unit and coronary intensive care unit, underwent 48 hours of TTM, were nontraumatic, and provided informed consent from their next of kin were included in the study.

Targeted temperature management application and data collection

A surface cooling system was used for TTM. A water-circulating surface cooling device and pads were prepared. Controlled cooling was achieved at an average rate of 0.25°C to 0.50°C per hour to reach a constant target temperature of 34°C within approximately four to six hours using water-circulating pads adhered to the trunk and lower extremities. After the desired TTM was applied for 48 hours, the patients underwent a re-warming process at an average rate of 0.25°C to 0.50°C per hour for approximately four to six hours. The device and cooling pads were removed from the patient when 72 hours had passed since the start of the initial application, at which point the patient's body temperature was within the normal range. Body temperatures were measured transesophageally in all patients, and their temperatures were checked hourly. All patients received midazolam (0.01-0.1 mg/kg/hour) and remifentanil (0.05-0.3 mcg/kg/minute) as needed for sedation and pain control. If neuromuscular blockade was required, rocuronium (0.2-0.4 mg/kg/hour) was used.

Venous blood samples were collected from patients who underwent TTM at 34°C for 48 hours at 0 (baseline), 12, 24, and 48 (termination) hours. After centrifugation at 3500 revolutions for 10 minutes, the serum components were transferred to Eppendorf tubes and stored at -80°C until the day of analysis. Demographic data of the patients included in the study, including CPR durations, Glasgow Coma Scale (GCS) scores on admission, the location of cardiac arrest, discharge status, performance of angiography, initial recorded heart rhythms, comorbidities, and length of hospital stay, were recorded. Informed consent was obtained from the patients' next of kin before venous blood collection. The collected serum samples were thawed on the day of analysis.

Analysis of TAS, TOS, OSI, and Nrf-2

The Nrf-2 level was analyzed using a commercially obtained enzyme-linked immunosorbent assay (ELISA) kit (BT-LAB) following the kit's protocol. In this protocol, a 96-well microplate pre-coated with Nrf-2 was used. Serum samples were added to the pre-coated 96-well plate and incubated. After washing to remove unbound molecules, biotinylated Nrf-2 was added to the wells to bind to the Nrf-2 in the samples. Subsequently, Streptavidin-HRP was added to bind to the biotinylated Nrf-2. After incubation, unbound streptavidin-HRP was washed away. Then, a substrate solution was added, and color development occurred proportionally to the amount of Nrf-2. The reaction was stopped by adding an acidic stop solution, and absorbance was measured at 450 nm using a microplate reader (Cytation-1, Biotek).

TAS levels were measured using commercially available kits (Relassay, Turkey). The new automated method is based on the bleaching of the characteristic color of the ABTS (2,2′-Azino-bis (3-ethylbenzothiazoline-6-sulfonic acid)) radical cation by antioxidants. The test has excellent precision values of less than 3%. Results were expressed as mmol trolox equivalent per liter (L) [9].

TOS levels were measured using commercially available kits (Relassay, Turkey). In the new method, oxidants present in the sample oxidized the iron ion-o-dianisidine complex to the iron ion. The oxidation reaction was enhanced by the abundance of glycerol molecules in the reaction medium. Ferrous ions formed a colored complex with xylene orange in an acidic environment. The color intensity, which could be measured spectrophotometrically, was related to the total amount of oxidant molecules in the sample. The analysis was calibrated with hydrogen peroxide, and the results were expressed as micromoles of hydrogen peroxide equivalent per liter (μmol H2O2 equivalent / L) [10].

The ratio of TOS to TAS was considered the oxidative stress index (OSI). For calculation, the resulting TAS unit was converted to μmol/L, and the OSI value was calculated using the following formula: OSI (arbitrary unit) = TOS (μmol H2O2 equivalent / L) / TAS (μmol trolox equivalent / L) [11].

Statistical analysis

Statistical analyses were performed using SPSS version 20 (IRB Inc., Armonk, New York). The normality of variables was assessed through visual methods (histograms and probability plots) and analytical techniques (Kolmogorov-Smirnov tests). Descriptive analyses were presented using the median and interquartile range for variables that did not follow a normal distribution. Since the distribution characteristics of TAS, TOS, OSI, and Nrf-2 variables did not meet the assumptions of parametric tests, the statistical significance of changes over time for these parameters was assessed using the Friedman test. If necessary, pairwise comparisons were conducted using the Wilcoxon test, and the results were adjusted using the Bonferroni correction. A total type-1 error level of 5% was used for statistical significance. A p-value of <0.05 was considered statistically significant.

## Results

Our study included 40 patients who were admitted with cardiac arrest and underwent TTM. Four patients who did not survive during TTM were excluded from the study, leaving a total of 36 patients to complete the research.

The demographic characteristics of the patients included in the study are summarized in Table 1 and Table 2. The average age of the patients participating in the study was 54.3±17.1 years. The mean duration of CPR was 16.1±9.5 minutes. The average hospital stay duration was found to be 22.3±27.0 days. The mean initial GCS score of the patients was 4.2±1.3.

**Table 1:** Descriptive characteristics of the patients CPR - cardiopulmonary resuscitation; GCS - Glasgow Coma Scale

Characteristics	Total number	Minimum	Maximum	Mean	Standard deviation
Age	36	18	75	54.3	17.1
CPR duration (min)	36	1	40	16.1	9.5
Length of hospital stay (days)	36	3	130	22.3	27.0
GCS	36	3	8	4.2	1.3

**Table 2:** Descriptive characteristics of the patients

Characteristics		Number	% percent
Gender	Male	25	69.4
	Female	11	30.6
	Total	36	100.0
Comorbidity	Yes	26	72.2
	No	10	27.8
	Total	36	100.0
Mortality	Exitus	26	72.2
	Discharged	10	27.8
	Total	36	100.0
Cardiac arrest location	Out of hospital	24	66.7
	In the hospital	12	33.3
	Total	36	100.0
Angiography status	No underwent angiography	26	72.2
	Underwent angiography	10	27.8
	Total	36	100.0
Initial determined heart rhythm	Non-shockable rhythm	25	69.4
	Shockable rhythm	11	30.6
	Total	36	100.0

It was determined that 69.4% of the patients participating in the study (25 patients) were male, while 30.6% (11 patients) were female. Among the patients, 72.2% (26 patients) had at least one chronic disease, including coronary artery disease, hypertension, diabetes, and chronic obstructive pulmonary disease, while 30.6% (11 patients) had no chronic diseases.

Among the patients, 72.2% (26 patients) in the intensive care unit did not survive, and 27.8% (10 patients) were discharged from the hospital. It was observed that 66.7% of the patients (24 patients) had cardiac arrest outside the hospital, while 33.3% (12 patients) had it inside the hospital. After admission, 27.8% of the patients (10 patients) underwent coronary angiography, as acute coronary syndrome was suspected by the cardiology department. Following monitoring and initiation of CPR, the first recorded heart rhythm was shockable in 30.6% of the patients (11 individuals; ventricular fibrillation, pulseless ventricular tachycardia), while it was non-shockable in 69.4% (25 individuals; asystole, pulseless electrical activity).

In the statistical analysis conducted, when the TAS and TOS measurements at 0 hours, 12 hours, 24 hours, and 48 hours were compared, it was observed that TAS and TOS levels gradually decreased throughout the TTM duration, with a statistically significant decrease found at the end of the 48th hour (p<0.05; Table 3).

**Table 3:** Changes in TAS, TOS, OSI, and Nrf-2 levels during targeted temperature management TAS - total antioxidant status, TOS - total oxidative stress, OSI - oxidative stress index; Nrf-2 - Nuclear factor erythroid 2-related factor 2

Measurements	TAS	TOS	OSI	Nrf-2
0 hours	0.9 ± 0.2	11.5 ± 3.1	1.3 ± 0.5	11.8 ± 4.6
12 hours	0.9 ± 0.2	11.3 ± 4.1	1.4 ± 0.6	12.0 ± 5.2
24 hours	0.8 ± 0.2	11.2 ± 3.7	1.4 ± 0.6	10.0 ± 6.2
48 hours	0.8 ± 0.2	9.5 ± 4.2	1.2 ± 0.5	10.5 ± 4.5
p-value	<0.005	0.026	0.452	0.169

In our statistical analysis, when OSI and Nrf-2 measurements were compared between hours 0, 12, 24, and 48, no significant difference was found between the hours (p>0.05; Table 3).

In our study, when patients who received CPR for less than 15 minutes and those who received CPR for more than 15 minutes were compared in terms of mortality, there was no statistically significant difference between the two groups (p>0.05). A total of 10 patients underwent coronary angiography, and PCI was performed on six of them. When patients who underwent coronary angiography and those who did not undergo it were compared in terms of mortality in our study, no statistically significant difference was found between the two groups (p>0.05).

## Discussion

The main findings of our study were that TTM application significantly reduced the TAS and TOS levels. However, there was no significant change in Nrf-2 or OSI levels during the TTM duration. 

Cardiac arrest is a global public health problem with a mortality rate exceeding 90%. After the ROSC following CA, approximately 40% of patients are admitted to intensive care units, while about 30% are discharged from the hospital. The three main reasons for this poor outcome are brain injury, myocardial dysfunction, and systemic ischemia/reperfusion injury, all of which are part of the pathophysiological process [12-15]. During circulatory arrest, cellular metabolism becomes anaerobic. As a result, intracellular acidosis occurs through the exchange of sodium and hydrogen ions. The excess intracellular sodium leads to an increase in calcium ions, causing secondary damage. Subsequently, the reperfusion phase exacerbates this damage. During CPR, mitochondrial dysfunction occurs due to the high intracellular oxygen pressure caused by high FiO2 ventilation, leading to increased production of reactive oxygen species. This results in oxidative damage to various cellular structures (phospholipids, proteins, and nucleic acids), disruption of cell function after ischemia, and the development of oxidative stress [16]. Reactive oxygen species (ROS) production dramatically increases with ROSC, and oxidative stress induced by ROS is recognized as having a central role in the development and progression of the pathophysiological process [17].

TTM is the only clinically proven treatment option to improve neurological outcomes associated with the pathophysiological process [18,19]. Among the neuroprotective mechanisms of TTM are the reduction of oxidative stress and anti-inflammatory effects [20-23]. TTM is considered one of the main components of post-cardiac arrest care. It is one of the evidence-based treatment methods applied for brain injuries occurring after resuscitation. TTM has many effects on pathways that become active, especially the antioxidant system, during ischemia-reperfusion injury [17].

There are studies in the literature evaluating oxidative stress and antioxidant levels in patients treated with TTM after CA. Clinical studies conducted on animal models have shown that TTM reduces oxidative stress. Leive et al. [24] demonstrated a decrease in cerebral cortex lipid peroxidation in dogs with ROSC after resuscitation, while Kuo et al. [25] showed a decrease in oxidative stress in rats subjected to brain cooling after traumatic brain injury. In a clinical study conducted by Fernanda et al. [26], it was found that the levels of malondialdehyde and protein carbonyl, which are used as markers of oxidative damage, decreased in patients treated with TTM. Concurrently, there was an increase in the activities of the erythrocyte antioxidant enzymes superoxide dismutase, glutathione peroxidase, and glutathione S-transferase, and a decrease in serum paraoxonase-1 activity was observed. In another study conducted by Xinyu et al. [27], oxidative stress parameters such as malondialdehyde and advanced oxidative protein products (AOPP) levels were compared with Superoxide Dismutase (SOD) levels in patients with acute cerebral infarction receiving TTM, showing a significant decrease in oxidative stress parameters in TTM-treated patients and a significant increase in SOD levels. In a study by Kenji et al. [7], oxidative stress markers diacron reactive oxygen metabolites (d-ROM) and antioxidant determinant biological antioxidant potential (BAP) levels were examined in patients receiving TTM after CA. When d-ROM and BAP were compared before and at the end of 24 hours of TTM application, a significant decrease in d-ROM and BAP levels was demonstrated.

Another method used for measuring oxidative stress in clinical settings is the measurement of TAS, TOS, and OSI. TAS represents the total antioxidants present in plasma and body fluids, and it is predicted to provide more accurate results than individual antioxidant measurements since antioxidants in the body interact with each other [9]. TOS, on the other hand, represents the total value of oxidative stress in the body. The measurement of TOS is considered more practical and valuable than examining individual oxidative stress parameters [10]. OSI is obtained by dividing TOS levels by TAS levels. This value is considered an indicator of the body's antioxidant response against oxidative stress to some extent. If the amount of free radicals exceeds the capacity of the endogenous antioxidant defense mechanism, oxidative stress occurs. The ratio of total oxidant status to total antioxidant status, known as OSI, serves as an indicator of the degree of oxidative damage. The elevation of oxidative parameters in atherosclerosis and the subsequent occurrence of acute myocardial infarction can be indicative of vascular damage. All these disturbances in the oxidant/antioxidant balances suggest that measurements in acute coronary syndrome diagnosis may serve as an alternative to long-term troponin monitoring [28]. Additionally, it has been shown that high OSI levels can predict unsuccessful CPR in CA patients [29].

There are limited studies regarding the use of TAS, TOS, and OSI in TTM. The closest study to ours among these studies is a study evaluating oxidative stress in 116 babies diagnosed with perinatal asphyxia after 72 hours of TTM, where TAS was found to be significantly higher (p=<0.001) after TTM. The study concluded that TTM reduces oxidative stress in perinatal asphyxia and improves neurological outcomes [30].

In our study, when TOS, which we used as an oxidative stress marker in patients undergoing TTM after CA, was compared at 0 hours (baseline), 12 hours, 24 hours, and 48 hours when TTM was discontinued, a gradual decrease in TOS levels was observed during TTM, but a statistically significant decrease was observed only at the end of 48 hours. The results of our study support findings from other studies (7,24-27).

As for TAS, which we used as an antioxidant level in our study, when compared at 0 hours (baseline), 12 hours, 24 hours, and 48 hours when TTM was discontinued, a gradual decrease in TAS levels was observed during TTM, but a statistically significant decrease was observed only at the end of 48 hours. There was no study found in the literature search that directly measured TAS levels. Contradictory findings exist in other clinical studies, with some showing low antioxidant levels and others showing high levels when one or more antioxidants were measured. TAS levels encompass all enzymatic and non-enzymatic antioxidants in the body. We anticipate that measuring TAS levels rather than individual antioxidant molecules may provide more accurate results. OSI, on the other hand, did not change significantly during TTM in our study.

NRF-2 is known to play a role in cellular defense by activating the transcription of antioxidant factors against oxidative stress and inflammation. It becomes active when oxidative stress increases and ROS are encountered. NRF-2 is the main regulator of glutathione and thioredoxin-dependent antioxidant enzymes (glutathione peroxidase, glutathione reductase, glutathione transferase, and thioredoxin reductase) [31,32].

In the literature, there is no study on Nrf-2 levels in patients undergoing TTM. Some studies related to Nrf-2 have shown an increase in Nrf-2 levels in cases where oxidative stress is increased. In a study by Masahiro et al. [33] it was demonstrated that the hyperactivation of the transcription factor Nrf2 is effective in suppressing oxidative stress resulting from ischemia-reperfusion injury and is protective against acute kidney damage. In a study conducted by Venkataramana et al. [34], it was found that Nrf-2 levels were significantly higher in smokers in response to oxidative stress caused by tobacco smoke compared to non-smokers.

In our study, there was no significant difference observed in Nrf-2 levels between the patients who received TTM after CA at 0 hours (when TTM started), 12 hours, 24 hours, and 48 hours (when TTM was discontinued) based on blood samples taken from the patients. It is known that Nrf-2 levels are induced by oxidative stress. The unchanged Nrf-2 levels in our study suggest that oxidative stress did not increase in the patients we treated with TTM. Besides, we found that total oxidative stress decreased after TTM therapy in the current study, and we therefore believe that Nrf-2 did not change accordingly. On the other hand, it should be noted that potential confounding variables that could affect antioxidant levels, such as comorbidities or medications, are not addressed in this study. We think that further studies with larger participants are required to determine the possible effect of comorbid diseases used medications on antioxidant levels.

Although our study is prospective, it may represent limited population diversity due to being limited to a single center. Additionally, the absence of a control group in the study may have weakened the statistical power of the study results. Moreover, we could have done our monitoring at more frequent intervals, but due to financial reasons, we had to keep the monitoring intervals a bit longer. Evaluation of the long-term outcomes of the patients who participated in the study will undoubtedly make an additional contribution to the study, but unfortunately, we could not follow up the patients long-term. 

## Conclusions

In conclusion, our study showed that the oxidant-antioxidant balance was maintained in patients who underwent target-oriented temperature treatment at 34°C for 48 hours after CA. Total oxidative stress gradually decreased at 0, 12, 24, and 48 hours, but a statistically significant decrease was observed only at the end of 48 hours. In line with this decrease, Nrf-2 levels, which are the key trigger for antioxidants in the body, did not change during the TTM. TAS levels also gradually decreased during the TTM, but a statistically significant decrease was observed only at the end of 48 hours. We anticipate that TTM after CA is effective on oxidative stress and antioxidant mechanisms and should be applied for at least 48 hours. We believe that there is a need for extensive, comprehensive, randomized controlled trials to better understand the effect of TTM on post-CA oxidative stress and antioxidant mechanisms.
